# The Impact of the “Osteo” Component of Osteosarcopenia on Fragility Fractures in Post-Menopausal Women

**DOI:** 10.3390/ijms22105256

**Published:** 2021-05-17

**Authors:** Yen-Huai Lin, Yu-Tai Shih, Michael Mu Huo Teng

**Affiliations:** 1 Department of Medical Imaging, Cheng Hsin General Hospital, Taipei 112, Taiwan; yhlin11@gmail.com (Y.-H.L.); n135724689@gmail.com (Y.-T.S.); 2 Department of Medicine, School of Medicine, National Yang-Ming University, Taipei 112, Taiwan; 3 Department of Medicine, School of Medicine, National Yang Ming Chiao Tung University, Taipei 112, Taiwan

**Keywords:** CHOS study, fragility fracture, osteoporosis, osteosarcopenia, trabecular bone score

## Abstract

Osteosarcopenia, the coexistence of bone and muscle loss, is common in older adults, but its definition lacks international consensus. This cross-sectional study (*n* = 1199 post-menopausal women) aimed to determine the association between osteosarcopenia and fragility fractures and to investigate the impact of the definition of the “osteo” component. Bone mineral density and bone microarchitecture were measured by dual-energy X-ray absorptiometry and the trabecular bone score (TBS), respectively. The “osteo” component of osteosarcopenia was classified as osteoporosis (T-score ≤ −2.5 SD), osteopenia/osteoporosis (T-score < −1 SD), and high-fracture-risk osteopenia (−2.5 SD < T-score < −1 SD)/osteoporosis (T-score ≤ −2.5 SD). The Fracture Risk Assessment Tool was used to identify high-fracture-risk osteopenia. Altogether, 30.3%, 32.2%, 14.4%, and 23.1% of participants had osteosarcopenia, osteoporosis alone, sarcopenia alone, and neither condition, respectively. The odds ratios between osteosarcopenia and fragility fractures were 3.70 (95% CI: 1.94–7.04) for osteosarcopenia, 2.48 (95% CI: 1.30–4.71) for osteoporosis alone, and 1.87 (95% CI: 0.84–4.14) for sarcopenia alone. Women with osteosarcopenia also had lower TBS, indicating worse bone microarchitecture. In conclusion, women with osteosarcopenia were more likely to have previously sustained a fracture compared to those without osteosarcopenia, with sarcopenia alone, and with osteoporosis alone. The relationship between osteosarcopenia and fracture risk may be best identified when considering high-fracture-risk osteopenia and osteoporosis.

## 1. Introduction

Osteosarcopenia is defined as the coexistence of bone and muscle loss, which is common in older adults. Both pathologies involve common risk factors, fracture risk, mortality rates, and healthcare costs [1,2,3,4,5,6]. Although the diagnostic criteria of osteoporosis and sarcopenia are well established [7,8,9], osteosarcopenia lacks international consensus on its definition. Yoo et al. defined osteosarcopenia as osteoporosis (T-score ≤ −2.5 standard deviation (SD)) and sarcopenia as defined by the Asian Working Group for Sarcopenia (AWGS) (low muscle strength and low muscle mass) [3]. Scott et al. defined osteosarcopenia as osteopenia/osteoporosis (T-score < −1 SD) and sarcopenia as defined by the European Working Group on Sarcopenia in Older People (EWGSOP) (low muscle mass and either low muscle strength or slow git speed) [10]. At present, there are limited studies regarding osteosarcopenia and its relationship to adverse events such as fractures.

In a longitudinal study of 1575 participants, patients with osteosarcopenia defined as osteopenia/osteoporosis (T-score < −1 SD) and sarcopenia by the EWGSOP definition had an increased radiographic fracture risk when compared to individuals without osteosarcopenia but not those with either condition [10]. Another longitudinal study of 1032participants, where osteosarcopenia was defined as osteopenia/osteoporosis (T-score < −1 SD) with either low muscle mass or muscle strength, showed similar results [11]. In a cross-sectional study of 253 participants, the risk of self-reported fractures was significantly higher in older adults with osteosarcopenia than in those without osteosarcopenia when using the sarcopenia definition of the Foundation for the National Institutes for Health (FNIH); however, when using the EWGSOP definition, the results were not significant [4]. These reports were preliminary and not conclusive. The heterogeneity in findings between studies was related to the inconsistent use of osteosarcopenia definitions. To the best of our knowledge, no study thus far has investigated the relationship between fractures and osteosarcopenia in accordance with the latest diagnostic criteria of the AWGS, published in 2019. In addition, the impact of the “osteo” component of osteosarcopenia has not been explored sufficiently.

Recently, the AWGS and EWGSOP updated the latest consensus on diagnostic algorithms of sarcopenia, and both introduced “possible or probable sarcopenia”, defined by either low muscle strength or reduced physical performance [8,9]. When diagnosed, the guidelines recommend the initiation of interventions. Therefore, the aim of this study was to determine the association between osteosarcopenia and fractures following the latest diagnostic criteria for possible sarcopenia from the AWGS in 2019 and to examine the impact of the definition of the “osteo” component of osteosarcopenia. We explored whether osteoporosis, osteopenia/osteoporosis, and high-risk osteopenia/osteoporosis had different associations with prevalent fractures and hypothesized that osteosarcopenia has higher prevalent fractures than either osteoporosis or sarcopenia.

## 2. Results

The characteristics of the study participants are shown in Table 1. In this study, 30.3% of participants had osteosarcopenia, 32.2% had osteoporosis alone, 14.4% had sarcopenia alone, and 23.1% had neither condition. In comparison to the other groups, women with osteosarcopenia had a higher prevalence of fractures, lower weight, lower grip strength, slower gait speed, and lower regional bone mineral density (BMD) scores and were older and shorter. In the neither-condition group, 12 participants took estrogen, 9 participants took glucocorticoids, and 1 participant took both estrogen and glucocorticoids.

Table 2 shows the results of the multivariable logistic regression analysis between osteosarcopenia and the prevalence of fragility fractures. The “osteo” component of osteosarcopenia was defined as osteoporosis (T-score ≤ −2.5 SD). The odds ratios for fragility fractures were 3.34 (95% confidence interval (CI): 1.92-5.81) for osteosarcopenia, 2.42 (95% CI: 1.38–4.23) for osteoporosis alone, and 2.15 (95% CI: 1.15–4.00) for sarcopenia alone. The fracture risk was higher in women with osteosarcopenia than in those with neither condition but not in those with either condition.

Table 3 shows the results of the multivariable logistic regression analysis where the “osteo” component of osteosarcopenia was defined as osteopenia/osteoporosis (T-score < −1 SD). The odds ratios for fragility fractures were 5.81 (95% CI: 0.76–44.3) for osteosarcopenia, 3.62 (95% CI: 0.48–27.6) for osteoporosis alone, and 4.86 (95% CI: 0.48–49.5) for sarcopenia alone. There was no significant risk of fractures under this definition of osteosarcopenia.

Table 4 shows the results of the multivariable logistic regression analysis where the “osteo” component of osteosarcopenia included high-risk osteopenia (−2.5 SD < T-score < −1 SD) and osteoporosis (T-score ≤ −2.5 SD). There were 115 osteopenic participants with a 10-year risk of major osteoporotic fracture ≥20% or risk of hip fracture ≥3%, who were considered as high-risk osteopenia. The odds ratios for fragility fractures were 3.70 (95% CI: 1.94–7.04) for osteosarcopenia, 2.48 (95% CI: 1.30–4.71) for osteoporosis alone, and 1.87 (95% CI: 0.84–4.14) for sarcopenia alone. Under this definition, individuals with osteosarcopenia had a higher prevalence of fractures than those without osteosarcopenia, with sarcopenia alone, and with osteoporosis alone.

Individuals with osteosarcopenia had a significantly lower trabecular bone score (TBS) than those without osteosarcopenia and those with sarcopenia alone (Table 5). Thus, individuals with osteosarcopenia had worse bone microarchitecture than those without osteosarcopenia and those with sarcopenia alone. The TBS according to fractures is shown in Supplementary Table S2.

## 3. Discussion

This study demonstrated that individuals with osteosarcopenia had higher prevalent fractures than those without osteosarcopenia, with sarcopenia alone, and with osteoporosis alone. In addition, differences in the definition of the “osteo” component of osteosarcopenia had an impact on the determination of fractures. The fracture was significant in groups for which the “osteo” component was defined as osteoporosis and high-risk osteopenia/osteoporosis, but it was not significant in the group for which the “osteo” component was defined as osteopenia/osteoporosis.

In the literature, individuals with the “sarco” component of osteosarcopenia as defined by the FNIH have a higher fracture risk than individuals without osteosarcopenia. [4]. Our study showed similar results when using the AWGS definition. While the definition for sarcopenia may not affect results related to fracture risk in individuals with osteosarcopenia, Sepúlveda-Loyola et al. reported that it may affect their physical performance and balance [4].

Osteosarcopenia lacks an international consensus on its definition, and the impact of the different definitions of the “osteo” component of osteosarcopenia must be demonstrated. In a cross-sectional study of 253 participants, Sepúlveda-Loyola et al. reported that the definition of the “osteo” component of osteosarcopenia did not affect the clinical outcomes [4]. However, in our study, differences in the definition had an impact on fracture risk. The risk was significantly higher in groups defined as having osteoporosis and high-risk osteopenia/osteoporosis, but it was not significant in the group defined as having osteopenia/osteoporosis (T-score < −1 SD). Additionally, the fracture risk was lower in the group defined as having osteoporosis (T-score ≤ −2.5 SD) than in the group defined as having high-risk osteopenia/osteoporosis because the reference group defined as having osteoporosis included those with high-risk osteopenia, thereby reasonably lowering the risk. Concerning fracture risk, most fractures occur in individuals with osteopenia [12]. From a public health perspective, it is important to identify individuals at risk of fracture to plan and perform early interventions. Therefore, we suggest that the definition of the “osteo” component of osteosarcopenia should include high-risk osteopenia and osteoporosis.

It is worth discussing whether osteosarcopenia increases fracture risk compared to the individual presence of either one of its components. In a longitudinal study of 1575 participants, those with osteosarcopenia defined as osteopenia/osteoporosis (T-score < −1 SD) and sarcopenia by the EWGSOP definition did not have an increased radiographic fracture risk compared to those with either condition [10]. In another longitudinal study of 1032 participants, those with osteosarcopenia, defined as osteopenia/osteoporosis (T-score < −1 SD) with either low muscle mass or muscle strength, did not have an increased self-reported fracture risk compared to those with either condition [11]. In our study, individuals with osteosarcopenia, as defined by the AWGS and high-risk osteopenia/osteoporosis, had an increased radiographic fracture risk when compared to individuals with sarcopenia alone and osteoporosis alone. The different definitions of osteosarcopenia affected the results.

This study had several strengths. First, to the best of our knowledge, this was the first study to investigate the effects of osteosarcopenia on prevalent fractures in women based on the latest diagnostic criteria of the AWGS, published in 2019. Second, to the best of our knowledge, this is the first study to demonstrate whether osteoporosis, osteopenia/osteoporosis, and high-risk osteopenia/osteoporosis have different associations with fractures. Third, this is the first study to investigate the bone microarchitecture based on the TBS in osteosarcopenia. However, this study also had some limitations. First, given that it had a cross-sectional design, causal relationships could not be inferred. Prospective studies will be required to confirm our results. Second, the “sarco” component of osteosarcopenia in this study was defined as low muscle strength rather than low muscle mass. However, the diagnostic algorithms of the AWGS and EWGSOP recommend the initiation of interventions in cases with low muscle strength. Third, this study only included Asian women, and the generalizability of the findings in men and other ethnicities may be limited. Fourth, calcium, vitamin D supplements, or other medications affecting bone metabolism were not investigated in this study. However, we included most medications that affect bone strength. Fifth, the physically and cognitively demanding nature of this study suggests that the generalizability of the findings to those who already have severe physical or cognitive disability should be examined further.

In conclusion, women with osteosarcopenia were more likely to have previously sustained a fracture compared to those without osteosarcopenia, with sarcopenia alone, and with osteoporosis alone. This relationship may be best identified when considering high-fracture-risk osteopenia and osteoporosis. Drug treatment should be initiated for these patients. Additionally, individuals with osteosarcopenia had worse bone microarchitecture than those without osteosarcopenia and those with sarcopenia alone.

## 4. Materials and Methods

### 4.1. Participants

The present study is part of the Cheng Hsin Osteoporosis and Sarcopenia (CHOS) study, which is a cross-sectional hospital-based study that has been ongoing since January 2019. The study design has been detailed elsewhere [13,14]. Post-menopausal Chinese women who had undergone dual-energy X-ray absorptiometry (DXA) examination were recruited, and only those who were willing and with a body mass index of 15–37 kg/m^2^ were included in this study. Well-trained project assistants conducted face-to-face interviews. Participants who could not complete the interview or were unable to perform either the hand grip strength or gait speed test because of severe physical or cognitive disability were not included in this study. Thus, 18 participants were excluded. Until September 2020, a total of 1199 participants were included, and written informed consent was obtained from each participant. This study was conducted following the rules of the Declaration of Helsinki of 1975, 2013 revision, and was approved by the institutional review board of Cheng Hsin General Hospital (IRB No. (660)107A-32).

### 4.2. Bone Mineral Density

We followed the 2019 International Society for Clinical Densitometry official positions [15]. The BMD at both the spine and hips was measured for all participants. In summary, we used L1-4 for the spine BMD measurement and excluded vertebrae that were affected by fracture or artifact. At least two vertebrae were used to measure the spine BMD. For the hip BMD, we measured both hips, and the site of any hip fracture was excluded from the analysis. The BMD at the lumbar spine, total hip, and femoral neck were assessed by a DXA scanner (Horizon W; Hologic Inc., Bedford, MA, USA). According to a World Health Organization report, a T-score ≤ −2.5 SD at any site indicates osteoporosis, whereas −2.5 SD < T-score < −1 SD at the lowest site indicates osteopenia [6]. Coefficients of variation of the BMD scores were 1.28% at the lumbar spine, 1.07% at the total hip, and 1.96% at the femoral neck.

### 4.3. Fracture Risk Assessment Tool (FRAX)

Calculations of major osteoporotic fractures and hip fractures were performed using Taiwanese BMD-adjusted FRAX online software (https://www.sheffield.ac.uk/FRAX/tool.aspx?country=26, accessed on 30 September 2020). The clinical risk factors were age, sex, weight, height, history of previous fracture, parental history of fractured hip, current smoking history, glucocorticoids, rheumatoid arthritis, secondary osteoporosis, alcohol consumption, and femoral neck BMD. According to the US National Osteoporosis Foundation (NOF), those with osteopenia (−2.5 SD < T-score < −1 SD) and a 10-year risk of major osteoporotic fracture ≥20% or risk of hip fracture ≥3% are considered a high-risk group, and treatment should be initiated [16]. The NOF guidelines are adopted by many Asian countries including Taiwan [17].

### 4.4. The “Sarco” Component of Osteosarcopenia

Hand grip strength (kg) of the dominant hand was measured using a digital dynamometer (EH101; Camry, Guangdong Province, China). The Camry dynamometer was validated with the Jamar dynamometer (Jamar, Jackson, MI, USA) [13,14]. Usual gait speed was measured on a 4-m course using a sensor timer. According to the AWGS 2019 consensus, the low muscle strength diagnostic cut-off value of hand grip strength was <18.0 kg for women, and reduced physical performance was defined as a gait speed <1.0 m/s [9]. Either low muscle strength or reduced physical performance was defined as possible sarcopenia in accordance with the diagnostic algorithm of the AWGS [9].

### 4.5. The “Osteo” Component of Osteosarcopenia

The “osteo” component of osteosarcopenia was classified as follows: (1) osteoporosis (T-score ≤ −2.5 SD), (2) osteopenia/osteoporosis (T-score < −1 SD), and (3) osteopenia (−2.5 SD < T-score < −1 SD), with high fracture risk based on the FRAX and osteoporosis (T-score ≤ −2.5 SD). The summary of the “osteo” component is shown in Supplementary Table S1.

### 4.6. Assessment of the Prevalence of Fragility Fractures

The prevalence of fragility fractures was assessed by retrospectively reviewing medical records of all participants. Only fractures confirmed by radiographic reports including spine, pelvis, or hip X-rays in the previous three years were included in this study. We included vertebral and hip fractures, and morphometric and incidentally noted vertebral fractures but excluded pathological fractures. In addition, our hospital is a regional hospital, and distal radial fractures are often treated in local medical facilities. Therefore, distal radial fractures were not included in this study.

### 4.7. Trabecular Bone Score

The TBS is a noninvasive analytical method based upon DXA images to evaluate bone microarchitecture [18]; it was measured using iNsight (version 3.0.2.0, Medimaps, Geneva, Switzerland). The coefficient of variation for the TBS was 2%.

### 4.8. Additional Covariates

The covariates included age (≥65 years vs. <65 years); body mass index (kg/m^2^); dairy product intake at more than 3 times per week (yes vs. no); regular exercise at more than 3 times per week (yes vs. no); the use of medications affecting bone strength, including either bisphosphonates, parathyroid hormone, or estrogen (yes vs. no); and the use of glucocorticoids (yes vs. no). The participants wore light clothing when weight and height were measured.

### 4.9. Statistical Analysis

The data were analyzed using SPSS Statistics for Windows, version 19.0 (IBM Corp., Armonk, NY, USA). The chi-square test and one-way analysis of variance were used to examine the factors related to osteosarcopenic categories. A stepwise multivariable logistic regression analysis was used to estimate the associations between osteosarcopenia and fragility fractures. A general linear model was used to compare the between-group differences in TBS with and without controlling for the effect of age. All reported *p*-values were two-sided, and *p* < 0.05 was considered statistically significant.

## Figures and Tables

**Table 1:** The characteristics according to osteosarcopenic categories (*n* = 1199), in which the “osteo” component was defined T-score ≤ −2.5 SD.

Variables	Neither Condition	Sarcopenia Alone	Osteoporosis Alone	Osteosarcopenia	
	*n* = 277	*n* = 173	*n* = 386	*n* = 363	*p* Value for χ2/One-Way ANOVA ^a^
Lifestyle factors (*n*, %)					
Regular exercise	97 (35.0)	48 (27.7)	126 (32.6)	117 (32.2)	0.459
Dairy product intake	98 (35.4)	55 (31.8)	126 (32.6)	132 (36.4)	0.619
Medications affecting bone strength (*n*, %)	13 (4.7)	15 (8.7)	58 (15.0)	49 (13.5)	<0.001
Glucocorticoids (*n*, %)	10 (3.6)	21 (12.1)	27 (7.0)	27 (7.2)	0.008
Prevalent fractures (*n*, %)					
Vertebral fracture	16 (5.8)	29 (16.8)	58 (15.0)	88 (24.2)	<0.001
Hip fracture	4 (1.4)	7 (4.0)	3 (0.8)	6 (1.7)	0.065 ^b^
Vertebral and hip fractures	19 (6.9)	34 (19.7)	60 (15.5)	91 (25.1)	<0.001
Age (years, mean ± SD)	60.3 ± 6.8	65.2 ± 8.1	63.6 ± 6.9	69.0 ± 7.9	<0.001
Anthropometric measurement (mean ± SD)					
Weight (kg)	60.6 ± 9.3	62.9 ± 11.2	55.4 ± 8.3	55.0 ± 8.7	<0.001
Height (cm)	157.7 ± 5.5	155.6 ± 5.7	155.8 ± 5.6	152.8 ± 5.5	<0.001
Body Mass Index (kg/m^2^)	24.4 ± 3.7	26.1 ± 4.6	22.8 ± 3.3	23.6 ± 3.6	<0.001
Hand grip strength (kg, mean ± SD)	24.1 ± 3.5	19.4 ± 4.8	23.3 ± 3.3	18.1 ± 4.2	<0.001
Gait speed (m/sec, mean ± SD)	1.3 ± 0.3	0.9 ± 0.2	1.3 ± 0.2	0.9 ± 0.2	<0.001
Bone mineral density (g/cm^2^, mean ± SD)					
Lumbar spine	0.919 ± 0.11	0.917 ± 0.12	0.728 ± 0.09	0.721 ± 0.11	<0.001
Femoral neck	0.684 ± 0.07	0.680 ± 0.08	0.565 ± 0.07	0.539 ± 0.07	<0.001
Total femur	0.827 ± 0.08	0.819 ± 0.10	0.697 ± 0.08	0.670 ± 0.08	<0.001

^a^: either χ2 test or one-way ANOVA test; ^b^: Fisher’s exact test.

**Table 2:** Odds ratios for fragility fractures according to osteosarcopenic categories, in which the “osteo” component was defined T-score ≤ −2.5 SD.

	Univariate		Stepwise Multivariable	
Variables	Odd Ratio	95% Confidence Interval	Odd Ratio	95% Confidence Interval
Osteosarcopenic categories				
Neither condition	1		1	
Sarcopenia alone	3.32	1.83–6.04	2.15	1.15–4.00
Osteoporosis alone	2.50	1.46–4.29	2.42	1.38–4.23
Osteosarcopenia	4.54	2.69–7.66	3.34	1.92–5.81
Age				
≥65 vs. <65	3.06	2.20–4.25	2.37	1.67–3.38
Body Mass Index	1.09	1.05–1.13	1.09	1.05–1.14
Glucocorticoids				
Yes vs. No	1.82	1.09–3.03	1.93	1.12–3.32
Medications affecting bone strength				
Yes vs. No	1.54	0.99–2.37		
Regular exercise				
Yes vs. No	0.92	0.67–1.28		
Dairy product intake				
Yes vs. No	0.83	0.60–1.15		

**Table 3:** Odds ratios for fragility fractures according to osteosarcopenic categories, in which the “osteo” component was defined T-score < −1 SD.

	Univariate		Stepwise Multivariable	
Variables	Odd Ratio	95% Confidence Interval	Odd Ratio	95% Confidence Interval
Osteosarcopenic categories				
Neither condition	1		1	
Sarcopenia alone	8.62	0.87–84.9	4.86	0.48–49.5
Osteoporosis alone	3.93	0.53–29.3	3.62	0.48–27.6
Osteosarcopenia	8.51	1.15–63.2	5.81	0.76–44.3
Age				
≥65 vs. <65	3.06	2.20–4.25	2.51	1.78–3.54
Body Mass Index	1.09	1.05–1.13	1.08	1.03–1.12
Glucocorticoids				
Yes vs. No	1.82	1.09–3.03	1.98	1.16–3.40
Medications affecting bone strength				
Yes vs. No	1.54	0.99–2.37		
Regular exercise				
Yes vs. No	0.92	0.67–1.28		
Dairy product intake				
Yes vs. No	0.83	0.60–1.15		

**Table 4:** Odds ratios for fragility fractures according to osteosarcopenic categories, in which the “osteo” component included high risk osteopenia (−2.5 SD < T-score < −1 SD) and osteoporosis (T-score ≤ −2.5 SD), using two different referents.

	Univariate		Stepwise Multivariable		Stepwise Multivariable	
Variables	Odd Ratio	95% Confidence Interval	Odd Ratio	95% Confidence Interval	Odd Ratio	95% Confidence Interval
Osteosarcopenic categories						
Neither condition	1		1		0.27	0.14-0.52
Sarcopenia alone	2.78	1.28–6.00	1.87	0.84–4.14	0.51	0.27–0.94
Osteoporosis alone	2.89	1.56–5.37	2.48	1.30–4.71	0.67	0.47–0.96
Osteosarcopenia	5.61	3.08–10.2	3.70	1.94–7.04	1	
Age						
≥65 vs. <65	3.06	2.20–4.25	2.18	1.51–3.13	2.18	1.51–3.13
Body Mass Index	1.09	1.05–1.13	1.09	1.05–1.14	1.09	1.05–1.14
Glucocorticoids						
Yes vs. No	1.82	1.09–3.03	1.79	1.04–3.08	1.79	1.04–3.08
Medications affecting bone strength						
Yes vs. No	1.54	0.99–2.37				
Regular exercise						
Yes vs. No	0.92	0.67–1.28				
Dairy product intake						
Yes vs. No	0.83	0.60–1.15				

**Table 5:** Trabecular bone score according to osteosarcopenic categories, in which the “osteo” component included high-risk osteopenia (−2.5 SD < T-score < −1 SD) and osteoporosis (T-score ≤ −2.5 SD).

	Trabecular Bone Score		
Osteosarcopenic Categories	Mean	Standard Deviation	*p*
Neither condition	1.317 ^c,d^	0.082	<0.001
Sarcopenia alone	1.311 ^c,d^	0.088	
Osteoporosis alone	1.254 ^a,b,d^	0.077	
Osteosarcopenia	1.240 ^a,b,c^	0.077	
	Estimated Marginal Mean ^e^	95% Confidence Interval	*p*
Neither condition	1.300 ^c,d^	1.289–1.311	<0.001
Sarcopenia alone	1.303 ^c,d^	1.288–1.317	
Osteoporosis alone	1.253 ^a,b^	1.245–1.260	
Osteosarcopenia	1.253 ^a,b^	1.245–1.261	

^a^ Significant difference to neither condition. ^b^ Significant difference to sarcopenia alone. ^c^ Significant difference to osteoporosis alone. ^d^ Significant difference to osteosarcopenia. ^e^ Estimates are age adjusted.

## Data Availability

The datasets used and analyzed during the current study are available from the corresponding author upon reasonable request.

## Supplementary Materials

The following are available online at https://www.mdpi.com/article/10.3390/ijms22105256/s1.
