# Supplementary material for: The Impact of the “Osteo” Component of Osteosarcopenia on Fragility Fractures in Post-Menopausal Women
Source: Int J Mol Sci. 2021 May 17;22(10):5256. doi: 10.3390/ijms22105256 (PMC8155869; doi:10.3390/ijms22105256)
Supplement: Supplementary file 1 [file ijms-22-05256-s001.zip › ijms-1178114-supplementary.pdf]

Supplementary Table S1. The summary of the "osteo" component of osteosarcopenia

The "osteo" component of osteosarcopenia associated with fractures	(1) osteoporosis (T-score ≤ −2.5 SD) shown in Table 2
	(2) osteopenia/osteoporosis (T-score < −1 SD) shown in Table 3
	(3) 1. osteopenia (−2.5 SD < T-score < −1 SD) with a 10-year risk of major osteoporotic fracture ≥ 20% or risk of hip fracture ≥ 3% based on the FRAX and<br><br>2. osteoporosis (T-score ≤ −2.5 SD) shown in Table 4

Supplementary Table S2. Trabecular bone score according to fractures

	Trabecular bone score		
	Mean	Standard deviation	*p*
Fracture			
Yes	1.239	0.080	<0.001
No	1.272	0.085	
